# The function and regulation of heat shock transcription factor in *Cryptococcus*

**DOI:** 10.3389/fcimb.2023.1195968

**Published:** 2023-04-24

**Authors:** Chenhao Suo, Yiru Gao, Chen Ding, Tianshu Sun

**Affiliations:** ^1^ College of Life and Health Sciences, Northeastern University, Shenyang, China; ^2^ Medical Research Center, State Key Laboratory of Complex Severe and Rare Diseases, Peking Union Medical College Hospital, Chinese Academy of Medical Science, Beijing, China; ^3^ Beijing Key Laboratory for Mechanisms Research and Precision Diagnosis of Invasive Fungal Diseases, Beijing, China

**Keywords:** heat shock factor, protein chaperone, *Cryptococcus*, fungal infection, thermotolerance

## Abstract

*Cryptococcus* species are opportunistic human fungal pathogens. Survival in a hostile environment, such as the elevated body temperatures of transmitting animals and humans, is crucial for *Cryptococcus* infection. Numerous intriguing investigations have shown that the Hsf family of thermotolerance transcription regulators plays a crucial role in the pathogen-host axis of *Cryptococcus*. Although Hsf1 is known to be a master regulator of the heat shock response through the activation of gene expression of heat shock proteins (Hsps). Hsf1 and other Hsfs are multifaceted transcription regulators that regulate the expression of genes involved in protein chaperones, metabolism, cell signal transduction, and the electron transfer chain. In *Saccharomyces cerevisiae*, a model organism, Hsf1’s working mechanism has been intensively examined. Nonetheless, the link between Hsfs and *Cryptococcus* pathogenicity remains poorly understood. This review will focus on the transcriptional regulation of Hsf function in *Cryptococcus*, as well as potential antifungal treatments targeting Hsf proteins.

## Introduction

Emerging and re-emerging fungal pathogens are one of the leading causes of human and animal illness and mortality (Akerfelt et al., 2010; Anckar and Sistonen, 2011; Brown et al., 2012; Fisher et al., 2012; Vos et al., 2012; Denning and Bromley, 2015; Gomez-Pastor et al., 2018). *Cryptococcus* species are encapsulated opportunistic pathogenic fungi that threaten human societies. Around 15% of AIDS-related deaths are caused by *Cryptococcus* infections, resulting in approximately 220,000 deaths per year (Idnurm et al., 2005; Kronstad et al., 2012; Erwig and Gow, 2016; Rajasingham et al., 2017). Recent researches have shown that infections caused by *Cryptococcus* species are a substantial public health concern in the Pacific Northwest of Northern America, Europe, Africa, and China (Byrnes et al., 2010; Byrnes et al., 2011; May et al., 2016; Liu et al., 2017). Nevertheless, antifungal medication options are limited, and the rapid development of drug resistance frequently impedes fungal treatment (Coste et al., 2004; Silver et al., 2004; Morschhauser et al., 2007; Dunkel et al., 2008; van der Linden et al., 2011; Kwon-Chung and Chang, 2012; Billmyre et al., 2020; Li et al., 2020; Priest et al., 2022).

**Figure 1:**
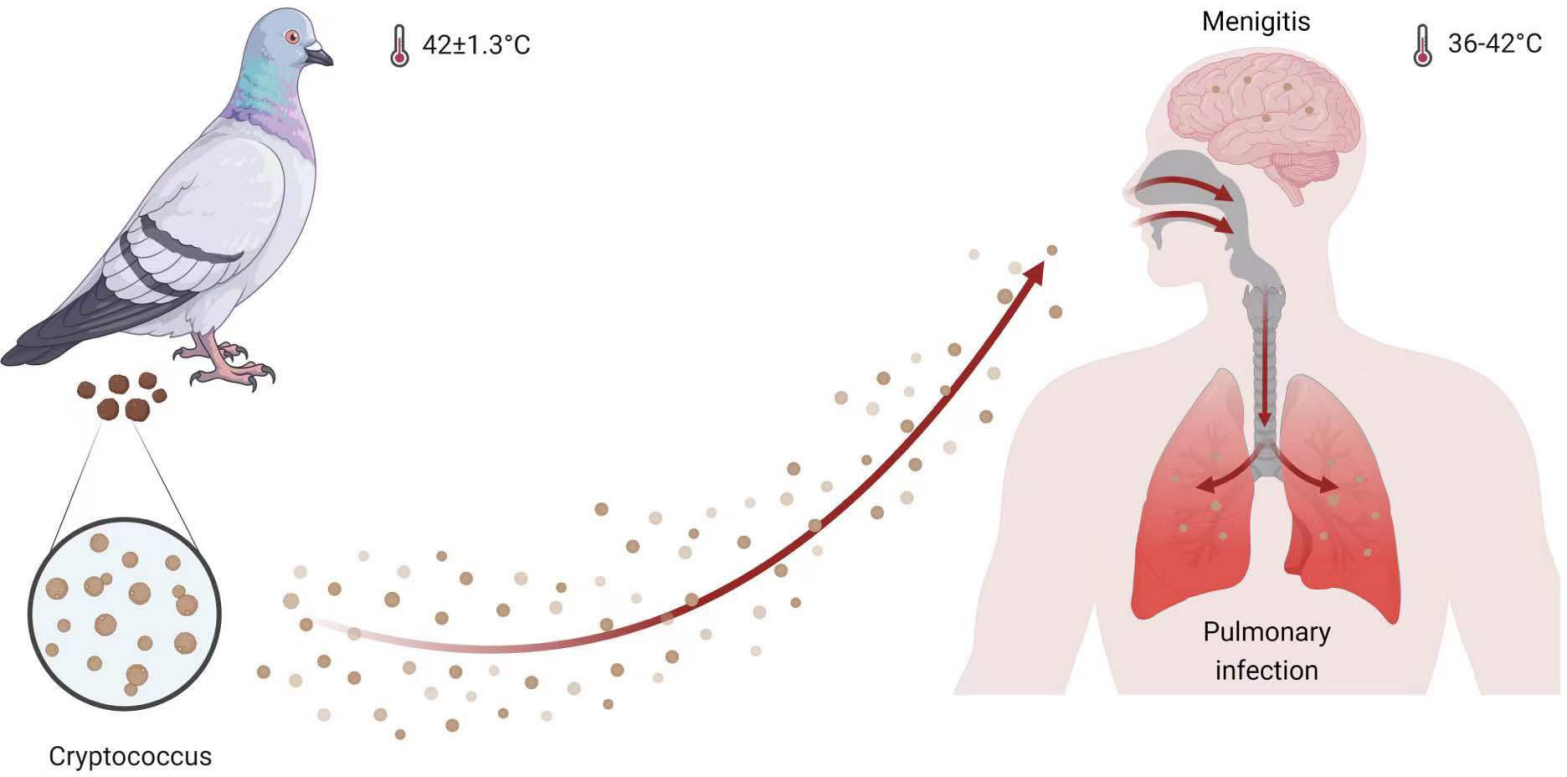
*Cryptococcus* infection model. Both humans and pigeons are hosts for *Cryptococcus* species, which specutilize thermotolerance to survive in pigeons with a body temperature of 42 ± 1.3°C and in humans with a temperature range of 36 to 42°C (Cramer et al., 2022). This figure was created with BioRender.com.

Infections caused by *Cryptococcus* begin in the lung tissue and subsequently spread to the central nervous system, resulting in fatal meningitis (Hull and Heitman, 2002; Kronstad et al., 2011; Kronstad et al., 2013). Machineries, such as those involved in morphological alterations, capsule formation, nutrition acquisition, and thermotolerance, play an essential role in regulating the colonization, invasion, and replication of cells in host tissues (Idnurm et al., 2005; Lee et al., 2013; Gerwien et al., 2018; Verbancic et al., 2018; Gao et al., 2022). The ability of *Cryptococcus* to survive at human body temperature of 37 °C as well as in even harsher temperature conditions, such as those found in the transmission animal species, such as pigeons, whose body temperatures are 42 ± 1.3°C (Adams et al., 1999), is a key factor that contributes to its success as a fungal pathogen (**Figure 1**). The heat shock transcription factor (Hsf) regulation axis is one of the most extensively studied systems controlling thermotolerance in eukaryotic cells, involving both canonical and non-canonical transcription regulation patterns (Akerfelt et al., 2010; Anckar and Sistonen, 2011; Mendillo et al., 2012; Gomez-Pastor et al., 2018; Veri et al., 2018). Numerous outstanding reviews provide a comprehensive overview of the relationship between Hsf activity and fungal biology (Yamamoto et al., 2007; Morano et al., 2012; Veri et al., 2018). Hence, this review focuses on the function of the Hsf family in *Cryptococcus* and elucidates its function and control in *Cryptococcus* pathogenicity.

## Canonical function of heat shock factor in *Cryptococcus*

**Figure 2:**
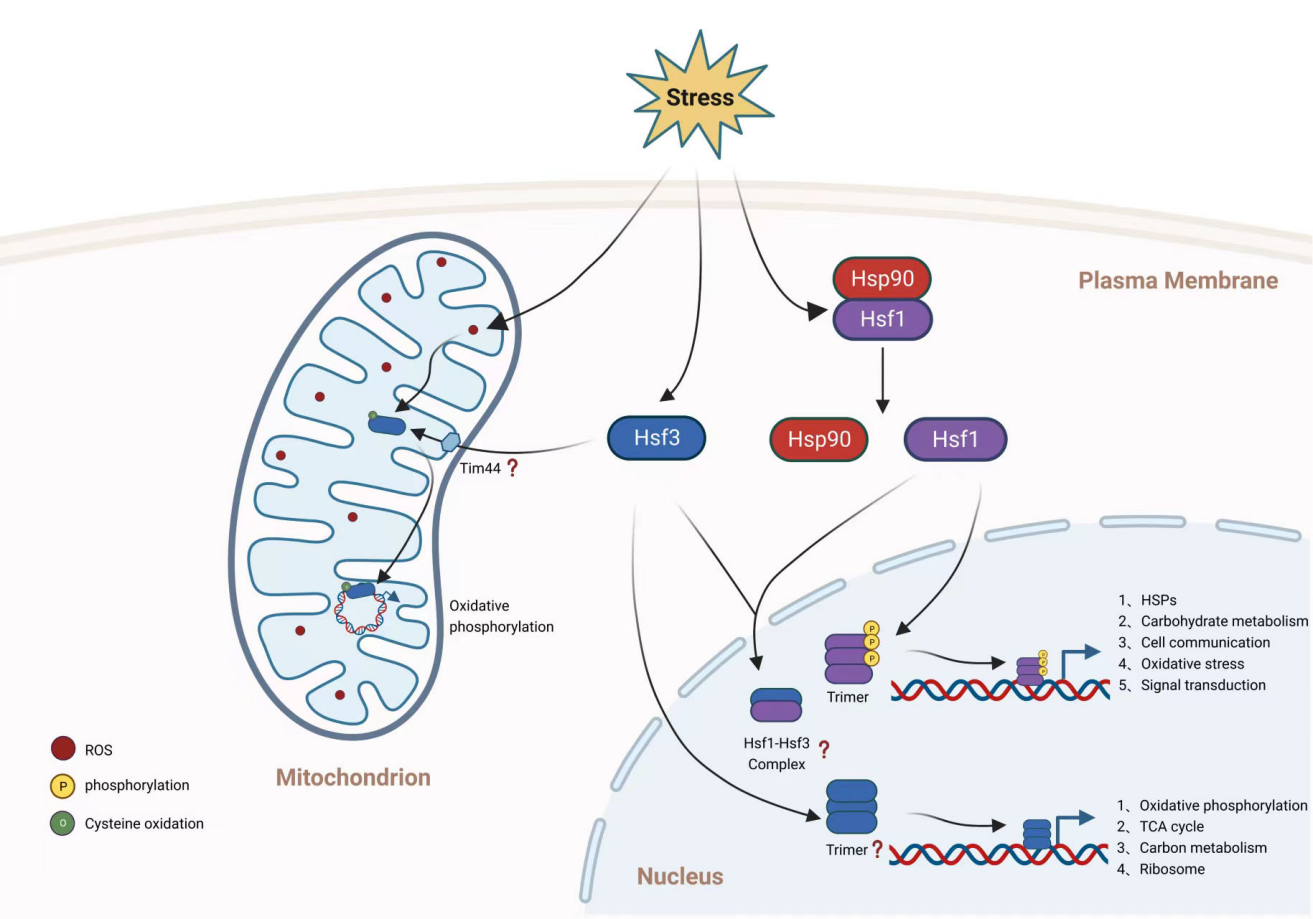
Hsf transcription regulation of *C. neoformans*. Hsf1 and Hsf3 participate in transcriptional regulation of gene expression, however the role of Hsf2 in *C. neoformans* is unknown. While Hsf1 and Hsf3 are transcription factors confined to the nucleus, Hsf3 is also translocated to the mitochondrion. As investigated in other organisms, Hsf1 in *C. neoformans* is likely repressed by Hsp90 under normal conditions and then translocated to the nucleus, where it is phosphorylated. The activated Hsf1 is subsequently trimerized to bind to gene promoter regions. Hsf1 of *C. neoformans* regulates the gene expression of canonical and noncanonical genes involved in glucose metabolism, cell communication, oxidative stress, and signal transduction. It is unknown if *C. neoformans* Hsf3 trimerizes in response to stimulation or if an Hsf1-Hsf3 complex is produced. Hsf3 in the nucleus of *C. neoformans* regulates the expression of genes involved in the TCA cycle, the electron transport chain, and the ribosome. ROS levels regulate the activity of mitochondrial Hsf3, which binds to the mitochondrial genome to promote the production of genes encoding the electron transport chain. This figure was created with BioRender.com.

Heat shock transcription factors (Hsfs) are a family of highly conserved DNA-binding proteins that provide thermoprotection to cells by activating the expression of canonical target genes encoding heat shock proteins (Hsps) (Akerfelt et al., 2010; Anckar and Sistonen, 2011). The activation of Hsf1 is dependent on an increase in temperature, which dissociates the Hsf1 inhibitory complex in the cytosol compartment to produce the DNA-binding-competent homotrimer complex (Akerfelt et al., 2010; Anckar and Sistonen, 2011) (**Figure 2**). Hsf1 complex binds heat shock element sequences of its target genes, which then activates downstream gene expression in response to intracellular stressors such as protein misfolding and oxidative damage. We now know that the Hsf family and its regulatory mechanism are highly conserved between *Saccharomyces cerevisiae* (which contains one *HSF* gene) and human cells, which contain six *HSF* genes (*HSF1*, *HSF2*, *HSF4*, *HSF5*, *HSFX* and *HSFY*) (Gomez-Pastor et al., 2018). Hsf1 is the most extensively studied of these Hsf transcription factors. Studies have indicated that Hsf1 is a crucial element in the development of human diseases, although little is known about its regulation in the production of fungal virulence factors (Neef et al., 2010; Neef et al., 2011; Neef et al., 2014; Gomez-Pastor et al., 2018; Dong et al., 2019; Dong et al., 2020). In mammals, for instance, the inhibition or elevation of Hsf1 results in the development of neurological disorders and cancer, respectively.

*C. neoformans HSF1*, similar to that of *S. cerevisiae*, is an essential gene for cell growth under normal conditions, as the *HSF1* endogenous promoter cannot be replaced with an inducible promoter (a galactose inducible promoter) without inhibiting cell development (Yang et al., 2017; Gao et al., 2022). Yang et al. studied the regulation mechanism of *C. neoformans HSF1* for the first time in a thermotolerance transcriptome analysis using DNA microarrays. They observed an unanticipated down-regulation of *HSF1* gene expression in response to elevated temperature. Another investigation of *HSF1* further supported this observation in *C. neoformans* (Gao et al., 2022). *HSP90* gene expression is strongly induced by temperature shifts despite the fact that *HSP90* has a conventional Hsf1 binding motif and is a direct target of Hsf1. Curiously, *HSF1* overexpression conferred tolerance to deadly temperature conditions (Yang et al., 2017). Why does *C. neoformans* inhibit the expression of the *HSF1* gene if it is essential for thermotolerance? In one scenario, the dramatic increase of Hsp90 inhibits *C. neoformans HSF1* gene expression in order to prevent its autoregulation of transcription (Gomez-Pastor et al., 2017). However, Hsf1 chromatin immunoprecipitation PCR analysis revealed no DNA amplification of its own promoter sequence, indicating that *C. neoformans HSF1* is regulated differently than in other fungal species (Yang et al., 2017).

In addition to regulating protein chaperones, a DNA microarray study revealed that *C. neoformans HSF1* acts as both a positive and negative transcriptional regulator in response to oxidative stress (Yang et al., 2017). In fact, a high-throughput ChIP sequencing investigation revealed that Hsf1 is a wide transcription regulator capable of DNA binding to noncanonical target genes involved in various biological processes, including glycolysis, cell communication, and signal transduction (Gao et al., 2022). The question of whether Hsf1 is a crucial virulence determinant remains unanswered, as the first experiment with an *HSF1* overexpression strain exhibited the same pathogenicity as the wild-type strain. However, given that *HSF1* is an essential gene, suppression of *C. neoformans HSF1* is likely a promising avenue for the development of anti-cryptococcal treatment.

In other organisms, particularly mammals, posttranslational modifications (PTM) such as phosphorylation, acetylation, and SUMOylation have been demonstrated to regulate Hsf1 activity (Westerheide et al., 2009; Asano et al., 2016; Hendriks et al., 2017). Fungal Hsf1 has been found to be phosphorylated (Hoj and Jakobsen, 1994; Liu and Thiele, 1996; Hashikawa and Sakurai, 2004), although additional PTM are still unknown (**Figure 2**). Hsf1 may not be acetylated in *C. neoformans*, according to a recent comprehensive acetylome investigation, which failed to detect Hsf1 acetylation (Li et al., 2019). In *S. cerevisiae*, the transition from a basal level of Hsf1 phosphorylation to a hyperphosphorylation process was assumed to constitute a regulation of Hsf1 transcriptional activity levels. When *C. neoformans* was exposed to a higher temperature, temporary phosphorylation of Hsf1 was also detected, likely indicating the activation of Hsf1 activity (Yang et al., 2017). Although direct phosphorylation enzymes to *C. neoformans* Hsf1 have not yet been identified, it has been demonstrated that a protein kinase, Sch9, regulates *HSF1* gene expression and protein level in a sophisticated way; that is, under basal conditions Sch9 regulates Hsf1 protein levels, whereas under heat shock conditions Sch9 represses *HSF1* gene expression. However, the PTM analysis of *C. neoformans* Hsf1 is greatly hindered by the lack of a thorough study of Hsf1 PTM utilizing pan-antibody coupled mass spectrometry analysis and the inadequacy of the production of specialized antibodies for Hsf1 PTM sites. Recently generated phosphatase and kinase knockout libraries provide key tools to facilitate and accelerate the identification of upstream PTM enzymes of *HSF1* and to map the regulation network of this essential transcription factor in *C. neoformans* (Lee et al., 2016; Jin et al., 2020).

## Noncanonical function of heat shock transcription factor in *Cryptococcus*

Despite the fact that Hsf1 is the master transcription regulator of canonical HSP gene expression in response to temperature elevation, Hsf1 also regulates a large number of noncanonical target genes (Mendillo et al., 2012; Gao et al., 2022). For example, in mammals, Hsf1 directly coordinates in regulation of gene expression of malignancy, including cell cycle regulation, signaling, metabolism and translation. In addition, other mammalian Hsfs, including human Hsf2, have been shown to coordinate with Hsf1 to form heterotrimer complexes that regulate the expression of noncanonical target genes involved in tumor progression and neurodevelopment (Shinkawa et al., 2011; Mendillo et al., 2012; El Fatimy et al., 2014; Jaeger et al., 2016).

According to the traditional paradigm of fungal Hsfs, fungal species contain only one copy of Hsf protein (Gomez-Pastor et al., 2018; Veri et al., 2018; Gao et al., 2022), particularly in the model yeast *S. cerevisiae* and *C. albicans*. *S. cerevisiae* may have deceived our knowledge of the evolution and transcriptional regulation of the Hsf family in fungi. However, a recent study identified at least two Hsf orthologs in the genomes of substantially all fungal species, although their roles in transcription regulation are unknown. Except for *S. cerevisiae* and *C. albicans*, all fungal species contain multiple copies of HSF genes. Protein sequence comparisons revealed that the preponderance of fungal pathogen Hsf1s have a high degree of similarity, whereas *C. neoformans* Hsf3 and Hsfs from other fungal species have a moderate degree of divergence. (Gao et al., 2022). In *C. neoformans*, expression of all three Hsfs, namely Hsf1, Hsf2 and Hsf3, are all responsive to temperature shift, with reciprocal pattern in transcription regulation; Hsf1 is downregulated under temperature elevation conditions, as Hsf2 and Hsf3 are induced in expression upon heat treatment. Loss of *C. neoformans HSF3* decreases the rate of cell survival under lethal temperature conditions and reduces fungal virulence and fitness in a mouse infection model (Gao et al., 2022). *C. neoformans* Hsf3 does not regulate gene expression of *HSPs*, instead binds directly to promoter regions of genes involved in carbohydrate metabolism (primarily genes involved in the tricarboxylic acid cycle, TCA) (**Figure 2**), and this resembles the noncanonical Hsf in mammals. However, *C. neoformans* Hsf1 and Hsf3 show high similarity in DNA-binding domain and share substantial overlap in target genes (mainly carbohydrate metabolic genes). Since it is now known that mammalian Hsf1 cooperates with other Hsfs, such as human Hsf2 (Jaeger et al., 2016; Gomez-Pastor et al., 2018), it is intriguing to examine whether *C. neoformans* Hsfs are regulated in a similar manner. Recent examination of mammalian Hsf2 revealed a transcription regulation in aerobic glycolysis (glycolysis) by interacting with euchromatic histone lysine methyltransferase 2 (EHMT2) to inhibit gene expression of fructose-bisphosphatase 1 (Fbp1) (Yang et al., 2019). Remarkably, *C. neoformans* promoter region of Fbp1 is concurrently bound by both *C. neoformans* Hsf1 and Hsf3. Five TCA intermediates, including citrate, isocitric acid, malate, fumarate, and alpha-ketoglutarate, are significantly induced when *C. neoformans HSF3* is absent.

Despite the fact that Hsfs are thought to be nucleus-localized transcription factors, *C. neoformans* Hsf3 was unexpectedly found in the fungal mitochondrial organelles, showing ubiquitous binding of Hsf3 to the mitochondrial DNA (Gao et al., 2022) (**Figure 2**). The induction of *HSF3* gene expression by mitochondrial stresses, such as heat and inhibitors of the mitochondrial complex, results in ROS overloads in *C. neoformans* mitochondria. Subsequently, intramitochondrial ROS oxidized Hsf3 to improve its ability to bind to DNA and activate the expression of downstream genes. How Hsf3 is recruited and translocated into the mitochondria of *C. neoformans* is unknown. It is probably via the universal mitochondrial translocase complex, TOM-TIM (Pfanner and Meijer, 1997). Protein co-IP followed by mass spectrometry identified the physical interaction between *C. neoformans* Hsf3 and Tim44 protein; however, further analysis is necessary to elucidate the mechanism of Hsf3 translocation. Results demonstrated that Hsf3 serves as a thermoprotector of *C. neoformans* via the regulation of gene expression of key components involved in electron transport chain, particularly the NDUFA5 subunit from complex I and Qcr9 subunit from complex III. Dampening Hsf3 protein level results in downregulation of ETC activity which promotes accumulation of intramitochondrial reactive oxygen species. The heat sensitive growth of the *HSF3* deletion *C. neoformans* strain is readily rescued by overexpression mitochondrial specific superoxide dismutase (*SOD2*). Furthermore, *C. neoformans* Hsf3 acts as a redox sensing protein via the oxidation of the 130th cysteine residues. The oxidation by reactive oxygen species improves *C. neoformans* Hsf3’s mitochondrial DNA binding affinity. In a separate study, mammalian Hsf2 was suggested to be the redox sensor that determines cell fate via its regulation axis of HSF2-BTG2-SOD2, in which ROS activation of mammalian Hsf2 triggers the gene expression of *BTG2* and *SOD2* (Kanugovi Vijayavittal et al., 2022). The major disparity between two sensing mechanisms is that *C. neoformans* Hsf3 does not control gene expression of *SOD2* (Gao et al., 2022). In addition, how does mammalian Hsf2 detect exclusive ROS? It is interesting to determine whether the cysteine residue at position 130 is conserved between two Hsfs.

## Hsfs as anti-cryptococcosis targets

Cryptococcosis has a significant death rate, yet treatment remains difficult due to the restricted number of antifungal drugs available (van der Linden et al., 2011; Billmyre et al., 2020; Li et al., 2020). In view of the therapeutic limitations, risks, and high costs associated with developing new antifungal medicines, the US Food and Drug Administration (FDA) has classified anti-cryptococcus medications as “orphan drugs,” offering regulatory support by decreasing the requirements for clinical research (Denning and Bromley, 2015). However, anticryptococcal medication resistance develops rapidly, surpassing the development of new therapeutic alternatives. Given that *C. neoformans* Hsf3 modulates virulence and Hsf1 is critical for regulating fungal growth (Yang et al., 2017), the Hsf family are prospective drug development targets. Given that the protein sequence of *C. neoformans* Hsf3 is similar to that of human Hsfs (Gao et al., 2022), it is necessary to develop inhibitors that target *C. neoformans* Hsfs but not human Hsfs (Gao et al., 2022). While the Hsf3 regulation axis may or may not be conserved in *Aspergillus* and *Candida*, Hsf1 is the highest priority option for therapeutical targets in the context of a larger understanding of fungal infections. Mammalian researches have offered new light on the production of HSF-targeting compounds for fungi (Neef et al., 2011; Neef et al., 2014; Dong et al., 2020). Mammalian Hsf1 activator and inhibitor have demonstrated efficacy in the treatment of mammalian ailments, such as neurological disorders and malignancies (Neef et al., 2011; Neef et al., 2014; Dong et al., 2020). For instance, a direct Hsf1 inhibitory chemical (Direct Targeted Hsf1 InhiBitor, DTHIB) that physically binds to human Hsf1 and induces its protein degradation suppresses the growth of tumors significantly (Dong et al., 2020). Given the significance of Hsf1 in *Cryptococcus*, it is intriguing to examine whether DTHIB can bind to and inhibit fungal proliferation *in vivo*.

## Conclusion and future directions

Fungal Hsf1 has been found and thoroughly characterized during the past thirty years, but our understanding of the regulation of the Hsf family in fungal virulence lags far behind. Not only have recent intriguing investigations of Hsfs from mammals and fungi revealed new regulatory mechanisms and potential therapeutical targets for infectious disease, but they have also inspired the microbial community to address more biological questions. The coordination relationship between *C. neoformans* Hsf1 and Hsf3, as well as the unexplored function of Hsf2, remain to be clarified, and a comprehensive regulation map of the *C. neoformans* Hsf regulation network is urgently required. The upshot could be the development of strategies for promoting the prevention of cryptococcosis and other invasive fungal infections.

## Author contributions

All authors contributed to the article and approved the submitted version. CS prepared the figure.
